# The Effect of Applying the Information-Motivation-Behavioral Skills Model on Treatment Adherence in Patients with Cardiovascular Disease: A Quasi-Experimental Study

**DOI:** 10.30476/ijcbnm.2021.88987.1563

**Published:** 2021-07

**Authors:** Nasrin Zahmatkeshan, Mahnaz Rakhshan, Ladan Zarshenas, Javad Kojuri, Zahra Khademian

**Affiliations:** 1 Department of Nursing, School of Nursing and Midwifery, Shiraz University of Medical Sciences, Shiraz, Iran; 2 Department of Nursing, School of Nursing and Midwifery, Community Based Psychiatric Care Research Center, Shiraz University of Medical Sciences, Shiraz, Iran; 3 Clinical Education Research Center, Shiraz University of Medical Sciences, Shiraz, Iran

**Keywords:** Cardiovascular diseases, Educational model, Medication adherence, Psychological model, Treatment adherence

## Abstract

**Background:**

Non-adherence complicates the management of patients with cardiovascular disease. This study aimed to determine the effect of applying the information-motivation-behavioral skills (IMB) model on the treatment adherence among these patients

**Methods:**

This quasi-experimental study was conducted on 112 patients with cardiovascular disease in Nemazee and Al-zahra hospitals in Shiraz, Iran, from October 2019 to July 2020. Eligible patients were selected and divided into intervention and control groups. The intervention was based on an integration of IMB model constructs and included 10 motivational-educational sessions for three months, followed by telephone consultations for six months. Data were collected before, and three and six months after the end of the motivational-educational sessions using adherence questionnaire in patients with chronic diseases, and adherence in chronic disease scale for medication adherence. Data were analyzed using SPSS 22, and descriptive statistics, chi-square, independent t-test, and repeated measure ANOVA were performed. P<0.05 was considered significant

**Results:**

The intervention and control groups were homogenous based on demographic characteristics. Repeated measure ANOVA findings revealed an increasing trend in the mean scores of the intervention group in treatment adherence from 51.10±3.20 at baseline to 66.40±5.50 three months and 73.80±6.80 six months after the end of the intervention (P<0.001). Furthermore, based on repeated measure ANOVA findings, the mean score of the intervention group in medication adherence significantly increased from 20.10+3 at baseline to 24.10+2.40 three months and 24.50+3.20 six months after the end of the intervention (P<0.001).

**Conclusion:**

Applying the IMB model promoted adherence to treatment and medication among patients with cardiovascular disease. Therefore, such interventions are recommended for these patients

## INTRODUCTION

Cardiovascular disease is highly prevalent worldwide. The disease had a prevalence of 523 million people in 2019 and accounted for 18.6 million deaths worldwide. ^1^ Evidence has indicated that more than 80% of cardiovascular disease mortalities occur in low- and middle-income countries. ^2^ In Iran, According to 2019 statistics, ischemic heart disease was the leading cause of death and the mortality rate in this disease was increasing, so that compared to 2009 this rate had increased by 29.90%. ^3^

Considering the chronic nature of cardiovascular disorders, patients are encountered with numerous problems in the long run. The prevalence of readmission was found to be 68.70% during the first year. ^4^ In Iran, the frequency of cardiovascular diseases readmission was 57%. ^5^ Non-adherence to treatment is one of the reasons for readmission in cardiovascular disease and poorer clinical outcomes, so that it has turned into a main challenge for medical and social sciences specialists. ^6^ On the other hand, improvement of adherence to treatment could promote the patients’ general health and quality of life, and their clinical outcomes. ^7^ In cardiovascular disorders, the prevalence of non-adherence to treatment was reported to vary from 40% to 60%. ^8 , 9^ In Iran, this value has been computed as 41%. ^10^ Evidence indicates the need to improve adherence among patients with cardiovascular disease. ^6 , 7^

Previous studies have investigated the effect of various interventions on medication adherence in patients with cardiovascular disease. Findings of a meta-analysis showed that providing information was an effective intervention to improve medication adherence among patients with cardiovascular disease. ^11^ Another review study showed that behavioral interventions had the greatest effects on the adherence to cardiovascular medicine in community settings. However, the information strategies showed to have no significant effects. ^12^ In addition, a review study revealed that behavioral multi-professional interventions could improve medication adherence; however, only a few studies with informational intervention directly investigated patient adherence, which did not show any significant effect on medication adherence. Therefore, it is suggested that the effectiveness of long-term multiple interventions on medication adherence in cardiovascular disease should be investigated. ^12^

Nurses have an important role in improving adherence to treatment and promoting the health of patients with cardiovascular disease. ^13^ To achieve the desired goals, nurses use knowledge rooted in nursing theories and models, ^14^ as well as knowledge derived from other disciplines as the basis of their research and practice. ^15^ One of the models used to improve adherence to treatment is the information-motivation-behavioral skills (IMB) model. The IMB model was a general social psychological conceptualization presented by Fisher and Fisher in 1992 to understand and improve the health behaviors. The assumption of the model is that the combination of three constructs including information, motivation and behavioral skills is the fundamental determinant of successful performance of health behaviors. According to the IMB model, information and motivation are often independent factors that primarily influence the adherence behavior through behavioral skills. ^16^ This model was first used for investigation of adherence to antiviral therapies in patients with human immunodeficiency virus, and its positive impacts on other chronic diseases including renal failure and diabetes were identified gradually. ^17 - 19^

The findings of a systematic review indicate that there is insufficient evidence for sustainability of behavioral changes after application of the IMB model for behavior change among patients with chronic disease, and further studies are needed to provide sufficient evidence in this regard. ^20^ In addition, an Iranian study which used IMB model in patients who had undergone coronary artery bypass graft indicated that while the model was effective on the patients’ information and motivation, there was no significant effect on behavioral skills. In the mentioned study, the intervention was a brief one-session intervention and its effect was evaluated after one month. However, the long-term effects of the intervention were not studied. ^21^ In addition, many studies on adherence to treatment in cardiovascular patients have considered only medication adherence. ^22 - 24^ Given that adherence to treatment in chronic diseases is a multidimensional and complex concept, ^25^ there should be a more comprehensive approach to adherence, so that other aspects beyond adherence to medication should be considered in studies in this field. Furthermore, although the IMB model has been used for determination of health behaviors for more than a decade, it has been applied less frequently among patients with cardiovascular disease. ^21^

Based on what was mentioned above, there is a need to study the effects of long-term interventions on the adherence to treatment, ^12^ and the need to a comprehensive approach toward the adherence to treatment has been identified. It has been proposed that the interventions based on models and theories were stronger and more influential in comparison to those that did not follow any specific framework. ^26 , 27^ However, there is a gap in the existing knowledge about the effectiveness of the models such as IMB on adherence to treatment in cardiovascular disease. Therefore, the present study aimed to determine the effect of applying the IMB model on the treatment adherence among the patients with cardiovascular disease.

## METHODS

This quasi-experimental study with pretest-posttest control design was conducted from October 2019 to July 2020 in Nemazee and Al-Zahra Heart Hospitals, as two main centers for hospitalization of patients with cardiac disease. The study population included all patients with cardiovascular disease hospitalized in coronary care units. Based on a similar study performed on the issue, ^28^ considering α=0.05 and power=0.8, σ_1_=9.37, σ_2_=13.28, and d=6.9, a 90-patient sample size was estimated for the study. However, this measure was increased to 112 considering the loss rate.

Sample size formula:

$n_{1}=n_{2}=\frac{{\left(Z_{1-\frac{\alpha }{2}}+Z_{1-\beta }\right)}^{2}(\sigma_{1}^{2}+\sigma_{2}^{2})}{d^{2}}$

$n_{1}=n_{2}=\frac{{\left(Z_{0.975}+Z_{0.8}\right)}^{2}{\left(9.37+13.28\right)}^{2}}{47.1}=45$

At the first, 112 eligible patients were selected and divided into intervention and control groups. The inclusion criteria of the study were age above 18 years, diagnosis of cardiovascular disease by a cardiologist, the ability to read and write, residence in Shiraz, lack of disabling ailments and cognitive disorders, and willingness to take part in the research. The exclusion criteria of the study were being absent in two or more intervention sessions, suffering from acute and unstable conditions, suffering chronic disorders unrelated to cardiovascular disease, and not responding to weekly telephone contacts. 

Both the intervention and control groups received the standard care programs presented by the treatment team, including periodical visits, prescription of medications, and diagnostic follow-ups throughout the investigation. The control group did not receive any other intervention. However, the patients in the intervention group were required to take part in a program designed based on the IMB model. This program was developed based on scientific resources in this area. Then, according the components of the IMB model, the program content was allocated to educational, motivational, and behavioral interventions in form of blue print. Afterwards, the sessions were held with seven experts, including three cardiologists, one cardiac rehabilitation specialist, and three individuals with PhD degrees in nursing, who were required to give their opinions, which were applied to the intervention program.

The final program incorporated the interventions based on three constructs of IMB model including informational, motivational and behavioral interventions, which are presented in Table 1. The program lasted for nine months and consisted of 10 educational-motivational sessions held for three months followed by telephone follow-ups for six months (held weekly during the first three months and every other week during the second three months). It should be noted that five weekly educational-motivational sessions were held in-person (three group sessions and two individual two-hour sessions), while the five others were telephone sessions. In the group sessions which were held at the school of nursing and midwifery, training programs were presented in homogeneous groups of 9-10 patients with similar problems. The content was presented through lectures, group discussions, short movies, educational pamphlets, and face-to-face training. Individual face to face sessions were held at the two heart clinics and were designed on the basis of the patients’ specific needs. During telephone sessions, the researcher contacted the patients via telephone on a weekly basis. One of the researchers (the first author) who was a PhD candidate in nursing and was experienced in taking care of cardiac patients was responsible for the interventions and follow-ups. 

**Table 1:** Contents of the interventions based on three constructs of the information-motivation-behavioral skills model

Constructs	Content presented during the study stages	
	Educational motivational sessions	Telephone follow-ups
Informational intervention	• Pathophysiology of cardiovascular disease and its risk factors	• The informational content was reviewed during telephone sessions.
	• Surgical therapies	• The learned information were assessed.
	• Nutrition	• The benefits of applying home care advices were explained.
	• Exercise and activity	
	• Smoking cessation	• Patients’ ambiguities about the disease and treatment measures were assessed and resolved.
	• Stress management	
	• Medication consumption and importance of adherence to treatment and risks of non-adherence	
Motivational intervention	• A two-hour motivational interview conducted in groups by a psychiatric nurse with MSc degree in order to enhance the patients’ motivation to change.	• During the follow ups, patients’ values and attitudes were sought.
	• The methods used for empowerment of motivation were applied in all training sessions included:	• Patients’ positive attitudes towards adherence to treatment were reinforced.
	o determining the patients’ motivations to change	• Benefits of adherence and costs of non-adherence were discussed.
	o encouraging the patient’s personal and social motivations to treatment adherence	• Patients’ confidence to treatment program and their readiness to change were explored.
	o encouraging patients’ self-reinforcement over time	• The change program was planned with the help of the patient.
	• The patients were guided through asking open questions, empathic listening, confirmation, and provision of feedback.	• The techniques such as asking open-ended questions and active listening were applied during follow ups to improve patients’ motivation to adherence.
Behavioral interventions	• Focused on the application of trainings in daily life	• During the follow-up period, the researcher assessed the patients’ execution of the intervention program and recorded the information in notebooks which its contents emphasized the patients’ performance as well as their progress in adherence to the guidelines and home care.
	• Specific techniques for integration of educational programs to daily activities were searched for each patient and emphasized	• Patients’ improvements were reinforced and if needed changes in activities was recommended during follow ups.
	• The patients’ adherence to the programs as well as their progress was explored	• The patients’ problems in implementing the intervention program at home were investigated.
	• Patients’ weak points were determined, and they were guided for better execution of the treatment program.	• Appropriate solutions to the patients’ problems with the help of patients themselves were sought.
		• The focus of the behavioral intervention was on the following areas:
		o examining how to apply the recommendations at home
		o how to control signs and symptoms (e.g. chest pain, dyspnea)
		o performing daily activities
		o the amount of exercise and activity and how to do it
		o assessing the process of quitting smoking
		o dietary management (asking the patient’s weekly diet)
		o stress management
		o patients’ using relaxation techniques
		o taking medications and how to control their side effects

The data were collected using a demographic information questionnaire, Adherence Questionnaire in Patients with Chronic Disease, ^25^ and a scale for medication adherence, i.e. Adherence in Chronic Disease Scale (ACDS). ^29^ Adherence Questionnaire in Patients with Chronic Disease was designed in the Iranian context by Sayyed Fatemi et al. and its psychometric properties were assessed in 2013. This questionnaire contained 40 items divided into seven dimensions, including making effort for treatment, intention to take the treatment, adaptability, integrating illness into life, sticking to treatment, commitment to treatment, and indecisiveness for applying treatment. The items could be responded via a five-point likert scale from completely disagree (1) to completely agree (5). The initial scores range from 40 to 200 that are converted to a range of zero to 100. Accordingly, scores 0-25, 26-49, 50-74, and 75-100 represented weak, moderate, good, and very good adherence to treatment, respectively. The content validity index (CVI) of the questionnaire was 0.91 and its reliability was confirmed by Cronbach’s alpha α=0.92. ^25^ This questionnaire has been used in another study in Iran. ^10^ In the present study, the content validity was reviewed by experts from nursing and medicine disciplines and the reliability of the tool was confirmed by Cronbach’s alpha α of 0.91.

ACDS was designed by Kubica et al. and contained seven items. The items were responded via a Likert scale from zero to four. As a result, the scores could range from zero to 28. Accordingly, scores below 20, between 21 and 26, and above 27 represented low, moderate, and high medication adherence, respectively. ^29^ This questionnaire was used in a study on adherence to treatment among patients with cardiovascular disease, and its reliability was confirmed by Cronbach’s alpha=0.75. ^30^ Since the reliability and validity of the Persian version of the questionnaire were not investigated before, translation, back-translation, and cultural adaptation were carried out in the present study. In order to determine the validity, qualitative face and content validity were determined. The results revealed content validity ratio of 0.89 and CVI of 0.94. Moreover, the reliability of the questionnaire was evaluated using the test-retest method and investigation of internal consistency. The results indicated intra-class correlation coefficient of 0.91 and α=0.85. The questionnaire was used after the confirmation of its psychometric properties. 

The study data were gathered prior to the intervention in the wards where the patients were hospitalized. Three months after the end of the educational-motivational sessions, all patients in both intervention and control groups completed the questionnaires in the cardiovascular clinics when they had referred for medical visits. Before the intervention and three months after the end of the intervention, the data were collected using paper questionnaires. However, six months after the end of the educational-motivational sessions, due to the spread of the Coronavirus, the same questionnaires were designed electronically and the data were collected after providing the respondents with sufficient information about completing them. Furthermore, two patients in the control group were excluded due to death. Therefore, at the end of the study, the data of 110 patients (56 in the intervention group and 54 in the control group) were analyzed.

This study was approved by the Ethics Committee of the University (code: IR.SUMS.REC.1398.575) as well as by the hospitals’ authorities. It should also be mentioned that the study objectives were explained to the patients and their written informed consent forms were obtained. To encourage the patients to continue participation in the study, free transportation services were provided and intervention sessions were adjusted based on their free time. 

The collected data were entered into the SPSS software, version 22. The variables were presented using descriptive statistics. Kolmogorov-Smirnov test showed normal distribution of treatment adherence (P=0.22) and medication adherence (P=0.13) data. Independent t-test was used to compare the two groups with respect to the means of quantitative variables. Besides, Chi-square test was employed to assess the relationship between the two qualitative variables. Moreover, repeated measure ANOVA was utilized in order to investigate the means of changes through the three stages of the intervention. To prevent the effect of time-group interaction on the findings, the data of treatment adherence and medication adherence were split according to the groups and repeated measure ANOVA was performed in each group separately. Therefore, the data within the group were reported separately and to compare between-groups data, independent t-test was used. Furthermore, Bonferroni post-hoc test was used to assess the changes in different stages of the research. Finally, Cohen’s d effect size for t-test was calculated for between-groups comparison of adherence to treatment and medication adherence mean scores.

## RESULTS

The results indicated that the mean age of the patients was 58.8±10.8 years. In addition, the mean ages of the intervention (57.1±11.0) and control groups (60.6±10.5) were matched (P=0.08). The majority of the patients were male (70.5%) and married (93.8%) and had middle school degrees (37.5%) (Table 2). Prior to the intervention, no significant difference was observed between the two groups in terms of demographic data and various dimensions of adherence to treatment and medication adherence (Tables 2 and 3).

**Table2:** Comparison of frequency distribution of demographic and clinical variables between the intervention and control groups

Groups	Intervention	Control	P value*
Demographic characteristics	N (%) (n=56)	N (%) (n=56)	
**Sex**			0.33
Male	41 (73.20)	38 (67.90)	
Female	15 (26.80)	18 (32.10)	
**Marital status**			0.13
Married	50 (89.30)	55 (98.20)	
Single	6 (10.70)	1 (1.80)	
**Education**			0.11
Primary School	12 (21.40)	14 (25)	
Guidance School	16 (28.60)	26 (46.40)	
High School	22 (39.30)	13 (23.20)	
Academic Education	6 (10.70)	3 (5.40)	
**Smoking History**			0.77
Cigar	17 (30.40)	15 (26.80)	
Waterpipe	3 (5.40)	5 (8.90)	
Opium	2 (3.60)	1 (1.80)	
Opium & Cigar	4 (7.10)	2 (3.60)	
None	30 (53.60)	33 (58.90)	
**Previous Admission History**			0.11
Once	13 (23.20)	18 (32.10)	
Twice	9 (16.10)	9 (16.10)	
Three Times	8 (14.30)	1 (1.80)	
More than Three	1 (1.80)	0 (0)	
None	25 (44.60)	28 (50)	
**Duration of Illness**			0.33
Less than 1 Year	10 (17.90)	8 (14.30)	
1 Year	21 (37.50)	27 (48.20)	
2 Years	10 (17.90)	14 (25)	
2-5 Years	4 (7.10)	2 (3.60)	
More than 5 Years	11 (19.50)	5 (8.90)	

* Chi square

**Table3:** Comparison of treatment adherence before and after the end of the intervention within and between groups

Variable	Time	Intervention Group	Control Group	P value*
		Mean±SD	Mean±SD	
Making effort for Treatment	Pre intervention	22.60±2.10	22.80±2.80	0.70
	3 Months after the intervention	31.10±2.80	24.60±3.10	0.018
	6 Months after the intervention	33.90±4.30	23.80±6.40	<0.001
P value**		<0.001	0.24	
Intention to take the Treatment	Pre intervention	18.40±2.10	18.50±1.70	0.76
	3 Months after the intervention	23.80±2.60	19.30±1.80	<0.001
	6 Months after the intervention	25.90±2.50	18.60±2.40	<0.001
P value**		<0.001	0.10	
Adaptability	Pre intervention	18.60±2.40	18.20±2.30	0.32
	3 Months after the intervention	23.50±2.40	18.90±2.80	<0.001
	6 Months after the intervention	25.10±2.50	18.60±1.30	<0.001
P value**		<0.001	0.30	
Integrating illness into life	Pre intervention	13.60±1.80	13.30±1.40	0.39
	3 Months after the intervention	14.46±2.07	11.70±1.70	<0.001
	6 Months after the intervention	18.48±1.91	11.50±1.20	<0.001
P value**		<0.001	0.013	
Sticking to Treatment	Pre intervention	9.10±1.50	9.60±1.10	0.07
	3 Months after the intervention	12.80±1.71	9.30±1.10	<0.001
	6 Months after the intervention	14.50±2.10	9.60±1.60	<0.001
P value**		0.001	0.26	
Commitment to Treatment	Pre intervention	12.60±1.90	12.10±1.70	0.14
	3 Months after the intervention	16.60±1.60	12.70±1.20	<0.001
	6 Months after the intervention	18.10±2.20	12.37±1.30	<0.001
P value**		<0.001	0.08	
Indecisiveness for applying Treatment	Pre intervention	7.10±1.30	6.80±1.30	0.16
	3 Months after the intervention	10.20±1.20	6.80±1.10	0.001
	6 Months after the intervention	10.70±1.10	6.40±1.10	<0.001
P value**		<0.001	0.06	
Total Score of Treatment Adherence^a^	Pre intervention	51.10±3.20	50.60±3.30	0.50
	3 Months after the intervention	66.40±5.50	51.90±3.80	<0.001
	6 Months after the intervention	73.80±6.80	52.40±6.10	<0.001
P value**		<0.001	0.12	
Medication Adherence	Pre intervention	20.10±3	19.20±4.20	0.16
	3 Months after the intervention	24.10±2.40	22.20±2.80	<0.001
	6 Months after the intervention	24.50±3.20	19.60±1.70	<0.001
P value**		<0.001)	0.23		

* Independent sample t-test;

** Repeated measure ANOVA;

a Total score of treatment adherence converted to a range of zero to 100.

The results of independent t-test revealed significantly higher total score of treatment adherence in the intervention group compared to the control group three and six months after the end of the intervention (P<0.001). Additionally, the mean score of medication adherence in the intervention group was significantly higher than the control group three and six months after the end of the intervention (P<0.001) (Table 3).

Repeated measure ANOVA findings revealed an increasing trend in the total mean scores of treatment adherence in the intervention group from 51.10±3.20 at baseline to 66.40±5.50 three months and 73.80±6.80 six months after the end of the intervention (P<0.001) (Table 3). The results of Bonferroni post-hoc test showed a significant difference between the mean scores at baseline and three months after the end of the intervention, those at baseline and six months after the end of the intervention, and those three and six months after the end of the intervention (P<0.001).

The results of repeated measure ANOVA indicated that in the control group, the total mean score of adherence to treatment was 50.60+3.30 before the intervention, which increased to 51.90+3.80 three months and 52.40+6.10 six months after the end of the intervention (P=0.12) (Table 3). The results of Bonferroni post-hoc test revealed no significant change in the overall score of adherence to treatment in the control group three months (P=0.25) and six months after the end of the intervention (P=0.24) in comparison to the baseline. However, based on Bonferroni post-hoc findings, improvement was observed in several dimensions of adherence, including making effort for treatment (P=0.01), intention to take the treatment (P=0.004), and adaptability (P<0.001), in the control group three months after the end of the intervention compared to the baseline. 

Based on repeated measure ANOVA findings, the mean score of medication adherence in the intervention group significantly increased three months (24.10±2.40) and six months (24.50±3.20) after the end of the intervention compared to the baseline (20.10±3) (Table 3). The results of Bonferroni post-hoc test revealed a significant difference between the mean scores of medication adherence in the intervention group at baseline and three and six months after the end of the intervention (P<0.001). However, no significant difference was observed between the mean scores obtained three and six months after the end of the intervention (P=0.17). 

Based on repeated measure ANOVA findings, in the control group, the mean score of medication adherence was 19.20±4.20 before the intervention, which increased to 22.20±2.80 three months after the end of the intervention and decreased to 19.60±1.70 six months after the end of the intervention (P=0.23) (Table 3). The results of Bonferroni post-hoc test demonstrated a significant difference between the mean scores of medication adherence in the control group before the intervention and three months after the end of the intervention (P<0.001). However, a significant decline was observed in the mean score of the control group six months after the end of the intervention compared to three months after the end of the intervention (P=0.004). A non-significant difference was also observed between the mean scores obtained prior to the intervention and six months after the end of the intervention (P=0.31). 

## DISCUSSION

The results indicated that applying the IMB model could improve the adherence to treatment among the patients with cardiovascular disease. The results revealed a significantly ascending trend in the mean scores of all dimensions of treatment adherence and medication adherence in the intervention group three and six months after the end of the intervention. Additionally, the effect size of the between-group comparisons indicated the clinical significance of the findings. Similarly, the results of a systematic review showed the positive effects of the interventions based on the IMB model on promotion of adherence to treatment among patients with chronic diseases. ^20^ Additionally, a research in South Korea indicated the effectiveness of this model in improvement of adherence to treatment among patients with type II diabetes during a three-month period. ^31^ Nonetheless, the studies conducted on the application of the IMB model in patients with cardiovascular disease revealed the effectiveness of this model on the knowledge and motivation for adherence to treatment, but not on the behavioral skills, ^21 , 28^ which was not consistent with the results of the present investigation. In this study, ascending trend in all dimensions of treatment adherence in the intervention group indicated an improvement in the patients’ performance. The difference between the results might be attributed to the design of the intervention in the current study. This study benefitted from a larger number of training sessions carried out for a longer time. Moreover, consultation follow-ups were continued for six months, which could play a pivotal role in promotion of the patients’ behavioral skills. This implies that in order to achieve a high adherence, trainings have to be continued in the long run. ^32^ It is necessary to note that understanding and recognizing the disease plays an important role in increasing adherence to treatment. ^8^ In addition, promoting the patients’ motivation is recognized as a necessary factor in treatment adherence. ^33^ In the present study, motivational educational content presented through establishing effective communication, strengthening and facilitating the care processes and feedbacks could be effective in promoting adherence to treatment among the patients. It is suggested that a good relationship between the patients and healthcare providers can improve adherence to treatment after discharge from the hospital. ^34^ Evidence has also confirmed the effectiveness of social supports on the part of the nurses and physicians in promotion of adherence to treatment after percutaneous coronary intervention. ^34^ Of course, the importance of the integration of informational, motivational and behavioral interventions in achieving the desired outcomes should not be overlooked. The findings demonstrate the long-term effectiveness of IMB model on adherence to treatment and medication among patients with cardiovascular disease. However, the existence of the IMB model does not eliminate the need for specific nursing models in improving treatment adherence in cardiovascular disease, and it is necessary for nurses to design nursing models in this field.

In the present study, significant positive changes were observed in some dimensions of adherence to treatment as well as in medication adherence in the control group three months after the end of the intervention. However, these changes were non-significant six months after the end of the intervention, and a significant decline was observed in some dimensions like ‘integrating illness to life’. It seems that patients make their best attempts to coordinate their treatments with their living conditions during the first days and weeks after discharge. Furthermore, they are more inclined to carry out more understandable therapeutic measures as well as those that are in line with their financial status. However, they tend to make less effort as time goes by. Acute condition after discharge is yet another factor that can affect drug consumption. In other words, medication adherence tends to decrease with the improvement in the patients’ condition. ^35^ In this respect, the World Health Organization announced that adherence to treatment was higher in patients with acute disorders compared to chronic disorders, but it was abandoned after six months. ^30^ For instance, adherence in patients with cardiovascular disease was high percentage immediately after discharge from the hospital, ^36^ while the consumption of statins was disrupted in forty to seventy five percent of the patients after six months. ^37^

In the present study, adherence to treatment at the time of hospitalization was low in both study grous. Previous studies have reported that the rate of adherence varies in chronic disorders like cardiovascular diseases. ^9^ Most of the patients in this study were older adults. Adherence to treatment in older people is lower than younger population. This is related to such factors as decreased abilities, dependence on others, and poor management of treatment due to physical problems. ^38 , 39^ Considering the fact that weak adherence to treatment and medications consumption can be accompanied by intensification of the symptoms and readmission, ^40^ this issue should be taken into account seriously among the patients with cardiac diseases. Hence, further studies are recommended to be conducted on improvement of adherence to treatment and prevention of complications and readmission.

In comparison to other studies conducted on the issue, the current study benefitted from several positive points. The first strong point of the study was the intervention. In fact, the interventions were designed by a coherent pattern on the basis of the patients’ needs for ten sessions. Another strong point of the research was the six-month follow-up in the intervention group, which was carried out weekly during the first three months and every other week within the second three months. Finally, yet importantly, various instruments were used for data collection in order to obtain more reliable results. As mentioned earlier, adherence questionnaire in patients with chronic disease and ACDS, which has been designed in the Iranian context, was utilized in this study. However, the study had some limitations, as well. The first study limitation was the fact that blinding was not possible due to the nature of the intervention because the patients were aware of their study groups. Another study limitation was the obligation to use online questionnaires six months after the end of the intervention due to the spread of the Coronavirus. Finally, although we excluded the individuals with chronic disorders unrelated to cardiovascular disease, we did not consider the number of medications that may affect their medication adherence.

## CONCLUSION

The findings demonstrated that applying the IMB model could improve the adherence to treatment and medication among the patients with cardiovascular disease. Therefore, nurses can play an important role in improving the patients’ treatment adherence through continuous training and follow ups, motivating patients, facilitating the care processes, and providing feedbacks. Providing these services requires constant nurse-patient communication which is possible in specialized community-based nursing clinics or through home nursing programs. Additionally, we recommend that nurse researchers investigate the effect of such interventions on the treatment adherence in other areas of community based nursing. In addition, there is a need for developing models specific to nursing explaining treatment adherence in cardiovascular disease. 
